# The Immune Response and Immunopathology of COVID-19

**DOI:** 10.3389/fimmu.2020.02037

**Published:** 2020-08-26

**Authors:** Esmaeil Mortaz, Payam Tabarsi, Mohammad Varahram, Gert Folkerts, Ian M. Adcock

**Affiliations:** ^1^Clinical Tuberculosis and Epidemiology Research Center, National Research Institute of Tuberculosis and Lung Diseases, Shahid Beheshti University of Medical Sciences, Tehran, Iran; ^2^Department of Immunology, School of Medicine, Shahid Beheshti University of Medical Sciences, Tehran, Iran; ^3^Mycobacteriology Research Center, National Research Institute of Tuberculosis and Lung Diseases (NRITLD), Masih Daneshvari Hospital, Shahid Beheshti University of Medical Sciences, Tehran, Iran; ^4^Division of Pharmacology, Faculty of Science, Utrecht Institute for Pharmaceutical Sciences, Utrecht University, Utrecht, Netherlands; ^5^Respiratory Section, Faculty of Medicine, National Heart and Lung Institute, Imperial College London, London, United Kingdom; ^6^Priority Research Centre for Asthma and Respiratory Diseases, Hunter Medical Research Institute, The University of Newcastle, Newcastle, NSW, Australia

**Keywords:** coronavirus, SARS-CoV-2, SARS-CoV, IL-6, pathogenesis, cytokines storm

## Abstract

Coronaviruses were first discovered in the 1960s and are named due to their crown-like shape. Sometimes, but not often, a coronavirus can infect both animals and humans. An acute respiratory disease, caused by a novel coronavirus (severe acute respiratory syndrome coronavirus-2 or SARS-CoV-2 previously known as 2019-nCoV) was identified as the cause of coronavirus disease 2019 (COVID-19) as it spread throughout China and subsequently across the globe. As of 14th July 2020, a total of 13.1 million confirmed cases globally and 572,426 deaths had been reported by the World Health Organization (WHO). SARS-CoV-2 belongs to the β-coronavirus family and shares extensive genomic identity with bat coronavirus suggesting that bats are the natural host. SARS-CoV-2 uses the same receptor, angiotensin-converting enzyme 2 (ACE2), as that for SARS-CoV, the coronavirus associated with the SARS outbreak in 2003. It mainly spreads through the respiratory tract with lymphopenia and cytokine storms occuring in the blood of subjects with severe disease. This suggests the existence of immunological dysregulation as an accompanying event during severe illness caused by this virus. The early recognition of this immunological phenotype could assist prompt recognition of patients who will progress to severe disease. Here we review the data of the immune response during COVID-19 infection. The current review summarizes our understanding of how immune dysregulation and altered cytokine networks contribute to the pathophysiology of COVID-19 patients.

## Introduction

In December 2019, a novel Coronavirus (nCoV), emerged in the Huanan wet food Market, where livestock animals are also traded, in Wuhan, Hubei Province in China. However, analysis of the first 41 hospitalized patients suggests that Wuhan seafood market may not be source of novel virus spreading (1).

This resulted in an epidemic of severe pneumonia of unknown cause (2). Genomic sequencing of viral isolates from five patients with pneumonia hospitalized from December 18 to December 29, 2019, indicated the presence of a previously unknown β-CoV strain in patients (3). This nCoV has subsequently spread from the site of the original outbreak in China and was named as SARS-CoV-2 by the World Health Organization (WHO) on January 12th 2020 and the disease as COVID-19 on 11th February 2020 (4). It was confirmed as having 75–80% resemblance to the coronavirus that caused severe acute respiratory syndrome (SARS-CoV) (5). COVID-19 currently affects 188 countries globally^1^ and up to July 14th 2020 the cumulative number of confirmed cases were 13.1 million people and at least 572,426 people have died with SARS-CoV-2 infection (6). The mortality rate varies from less than 1% up to 3.7% between countries (7) compared with a mortality rate of less than 0.1% from influenza^2^. Given the origin of the first case of COVID-19, the infection was probably transmitted from animal to human.

Coronaviruses have caused three epidemics in the past two decades namely, COVID-19, SARS, and Middle East respiratory syndrome (MERS) (8). No specific antiviral therapies currently exist but efforts to develop anti-viral therapies and a vaccine are urgently needed. This review summarizes the immune response against SARS-CoV-2 and indicates areas of interest for the development of specific anti-viral therapies against SARS-CoV-2.

## Coronavirus

CoV belong to the genus Coronavirus in the *Coronaviridae* family. CoVs are pleomorphic RNA viruses with special crown-shape peplomers between 80 and 160 nM in size and a genome of 27–32 kb (8). Thus, enveloped CoV are some of the largest known RNA viruses (9, 10). Coronaviruses are able to infect a variety of hosts such as humans and several other vertebrates. They are associated with several respiratory and intestinal tract infections. Pulmonary coronaviruses have long been recognized as harmful pathogens in domesticated animals that also cause upper respiratory tract infections in humans (11).

Four coronavirus genera (α, β, γ, and δ) have been characterized so far, with human coronaviruses (HCoVs) detected as being in either the α (HCoV-229E and NL63) or β (MERS-CoV, SARS-CoV, HCoV-OC43, and HCoV-HKU1) genera (12). *Coronaviruses* have a high mutation rate and a high capacity to act as pathogens when present in humans and various animals presenting with a wide range of clinical features. The disease characteristics can range from an asymptomatic course to the requirement of hospitalization in an intensive care unit. *Coronaviruses* cause infections of the respiratory, gastrointestinal, hepatic, heart, renal and neurologic systems and exacerbations of lung diseases, croup and bronchiolitis (12–23).

Coronaviruses were not considered as highly pathogenic for humans until the outbreak of SARS in 2002–2003. Before these outbreaks the two most well-known types of CoV were CoV OC43 and CoV 229E that induced mild infections in immunocompromised individuals (13, 24, 25). Furthermore, 10 years after the SARS epidemic, another highly pathogenic CoV, MERS-CoV emerged in Middle Eastern countries (2).

## Angiotensin-Converting Enzyme 2 (ACE2)

Angiotensin converting enzyme (ACE) catalyses the formation of angiotensin II from angiotensin I and, thereby, plays a key role in the control of cardio-renal function and blood pressure (26). ACE is highly expressed in the human heart, kidney, and testis consistent with its role in cardio-renal function. ACE2 is a novel gene encoding a homolog of ACE (27) that efficiently cleaves the C-terminal residue from several peptides unrelated to the renin–angiotensin system (28). Although highest ACE2 mRNA expression levels were detected in the intestinal epithelium, pulmonary ACE2 expression and function have been given extensive attention in recent years due to the findings that ACE2 serves as the receptor for SARS-CoV (29, 30) and its role in acute lung injury (31). ACE2 expression within bronchial and nasal epithelial cells is mostly localized to goblet and mucociliary cells (30). Recent evidence shows that cell entry of SARS-CoV-2 via ACE2 could be inhibited by a pharmacologic inhibitor of the cellular serine protease TMPRSS2, which is employed by SARS-CoV-2 for S protein priming (32).

Angiotensin-converting enzyme 2 acts as a binding site or receptor for the viral anchoring or spike (S) proteins present on the exterior surfaces of beta coronaviruses (33). Upon viral binding, ACE2 is released from the epithelial cell surface into the airway surface liquid (34) via cleavage by ADAM metallopeptidase domain 17 (ADAM17) and other sheddases (35, 36). ADAM17 activation also processes the membrane form of the interleukin (IL)-6 receptor (IL-6R)-α to the soluble form (sIL-6Ra) allowing gp130-mediated activation of the transcription factor STAT3 (signal transducer and activator of transcription 3) via an sIL-6Ra-IL-6 complex in a variety of IL-6R-α-negative non-immune cells including airway epithelial cells (37). STAT3 activation, in turn, induces full activation of the pro-inflammatory nuclear factor kappa B (NF-κB) pathway (37). Thus, SARS-CoV-2 infection in the respiratory tract can activate both NF-κB and STAT3 in a feedforward mechanism (IL-6 amplifier or IL-6 Amp) leading to multiple inflammatory and autoimmune diseases (37). Since IL-6 is a functional marker of cellular senescence, the age-dependent enhancement of the IL-6 Amp might correspond to the age-dependent increase in COVID-19 mortality. Furthermore, the putative driving role of IL-6 in SARS-CoV-2 induced inflammation suggests that inhibition of Janus kinases may be an attractive therapy for severe COVID-19 patients (38).

Airway surface liquid can contain catalytically active shed or soluble ACE2 (sACE2) under both stimulated and constitutive conditions (39). sACE2 acts in a feedback loop to suppress viral entry into cells and suggests that reductions in ACE2 shedding might contribute to disease pathogenesis (40).

Modulation of ACE2 expression is seen in many lung diseases including acute lung injury (ALI). ALI is induced by viral and bacterial infections and by gastro-intestinal events such as diarrhea SARS infection induces ALI following binding to airway epithelial cells, it is known that as the virus binds to ACEs, the abundance on the cell surface, mRNA expression, and the enzymatic activity of ACE2 are significantly reduced due shedding/internalizing processes (41, 42). Interestingly in an animal model of SARS infection, binding of the virus to ACE2 results in decreased receptor expression and severe enhancement of acid aspiration pneumonia (43). Downregulation of ACE2 following SARS infection upregulates angiotensin (Ang) II which leads, in turn, to enhanced vessels permeability and induces lung injury (43). Importantly, ACE2 is endocytosed together with SARS-CoV, resulting in the reduction of ACE2 on cells, followed by an increase of serum Ang II (44). Severe lung inflammation itself may induce dysregulation of the renin-angiotensin pathway followed by ARDS development following SARS-CoV-2 infection. Indeed, SARS-CoV-induced ARDS in an animal model is prevented by inhibitors of angiotensin receptor type 1 (AT1R) (44).

Angiotensin-converting enzyme 2 is also implicated in the pathogenesis of lung fibrosis as it modulates neutrophil infiltration in the lung by inhibiting the Ang II/AT1R axis, triggering lung fibrosis (45). In addition, the expression of Ang 1–7, an ACE2-mediated anti-inflammatory metabolite of Ang II, is dysregulated in asthma suggesting a role in asthma pathogenesis (5, 46). In addition, the expression of ACE2 is down-regulated by the asthma-associated cytokine IL-13 which may account for the lower expression of ACE2 in nasal epithelial cells of asthmatic subjects (47).

Human ACE2 is the receptor for SARS- CoV (48) as well as for SARS-CoV-2 (3, 49). The binding of the SARS-CoV-2 viral S protein appears not to be as strong as that seen with the SARS virus (3). However, other studies suggest that the SARS-CoV-2 receptor binding domain (RBD) exhibits a significantly higher binding affinity for ACE2 than SARS-CoV RBD (50). Furthermore, additional reports suggest that receptors such as dipeptidyl peptidase 4 (DPP4 or CD26), which are involved in SARS and MERS infection, may also be important in SARS-CoV-2 infection (51–54).

## Immunopathology of COVID-19 Disease

The pathogenesis of COVID-19 is not defined but reports from many countries indicate that the virus has the same mechanism by which it enters or invades host cells as SARS-COV. The origin of SARS-CoV-2 is not well-established, however, it is established that bats are the source of related viruses and that human to human transmission plays a critical role in its pathogenesis (1, 49, 55, 56). After entering into target cells following Spike protein association with its receptor (57), viral RNA is encapsulated and polyadenylated, and encodes various structural and non-structural polypeptide genes. These polyproteins are cleaved by proteases that exhibit chymotrypsin-like activity (58, 59). Although transmembrane serine protease 2 (TMPRSS2) is the major protease associated with CoV activation and has been linked to SARS-CoV-2 activation, recent evidence from single cell RNA-sequencing (scRNA-seq) analysis shows that ACE2 and TMPRSS2 are not expressed in the same cell (30) suggesting the involvement of other proteases such as cathepsin B and L in this process.

**FIGURE 1:**
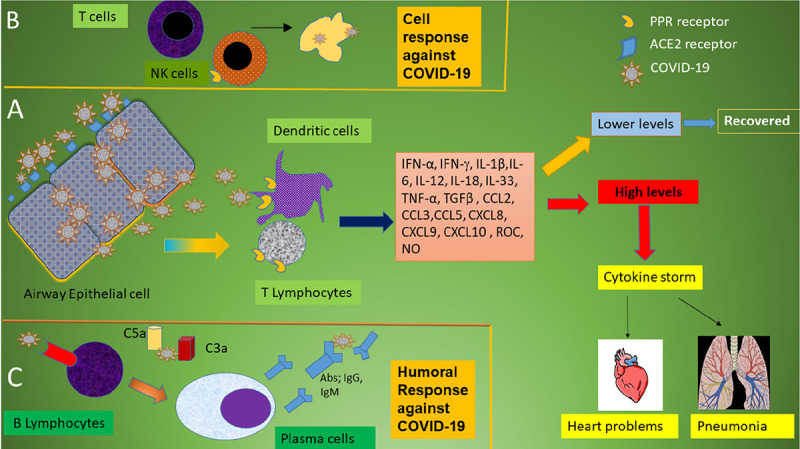
Schematic immune responses to CoVs. **(A)** When the SARS-CoV-2 virus invades the host, it is first recognized by the angiotensin converting enzyme (ACE) 2 receptor present on respiratory epithelial cells allowing viral entry. Following viral replication within the cells, the virus is released where it is met by the host’s innate immune system. T lymphocytes and dendritic cells are activated through pattern recognition receptors (PRRs) including C-type lectin-like receptors, Toll-like receptor (TLR), NOD-like receptor (NLR), and RIG-I-like receptor (RLR). The virus induces the expression of numerous inflammatory factors, maturation of dendritic cells, and the synthesis of type I interferons (IFNs) which limits the viral spread and accelerates macrophage phagocytosis of viral antigens resulting in clinical recovery. However, the N protein of SARS-CoV can help the virus escape from the immune responses and overreaction of the immune system generates high levels of inflammatory mediators and free radicals. These induce severe local damage to the lungs and other organs, and, in the worst scenario, multi-organ failure and even death. **(B)** The adaptive immune response joins the fight against the virus. T lymphocytes including CD4 + and CD8 + T cells play an important role in this defense. CD4 + T cells stimulate B cells to produce virus-specific antibodies whilst CD8 + T cells are able to directly kill virus-infected cells. T helper cells produce pro-inflammatory cytokines to help the defending cells. However, SARS-CoV-2 can inhibit T cells by inducing programmed cell death (apoptosis). **(C)** Humoral immunity including complement factors such as C3a and C5a and specific B cell-derived antibodies are also essential in combating SARS-CoV-2 infection.

In general, pattern recognition receptors (PRRs) recognize invading pathogens including viruses (60). Viruses elicit several key host immune responses such as increasing the release of inflammatory factors, induction and maturation of dendritic cells (DCs) and increasing the synthesis of type I interferons (IFNs), which are important in limiting viral spread (60). Both the innate and acquired immune response are activated by SARS-CoV-2. CD4 + T cells stimulate B cells to produce virus-specific antibodies including immunoglobulin (Ig)G and IgM and CD8 + T cells directly kill virus-infected cells (Figure 1). T helper cells produce pro-inflammatory cytokines and mediators to help the other immune cells. SARS-CoV-2 can block the host immune defense by suppressing T cell functions by inducing their programmed cell death e.g., by apoptosis. Furthermore, the host production of complement factors such as C3a and C5a and antibodies are critical in combating the viral infection (Figure 1) (61–64).

Viral-Track is a novel computational approach that screens scRNA-seq data for viral RNAs (65). This approach identified a major change in the bronchoalveolar lavage immune cell landscape during severe SARS-CoV-2 infection. Interestingly, Viral–Track identified co-infection of monocytes with human metapneumovirus following dampening of the IFN response.

The pathogenesis of COVID-19 is therefore as much a result of an abnormal host response or overreaction of the immune system in some patients with unknown etiology. This results in the local production of extremely high levels of a large number of inflammatory cytokines, chemokines and free radicals locally that cause severe damage to the lungs and other organs. In the worst-case scenario, systemic overspill results in multi-organ failure and even death (66, 67). Acute respiratory distress syndrome (ARDS) is the main death cause in COVID-19 (1). However, the precise reason for this being the common immunopathological event for SARS-CoV-2, SARS-CoV, and MERS-CoV infections is unclear although it probably involves the generation of a cytokine storm (68). COVID-19 infection induces pneumonia which is characterized primarily by fever, cough, dyspnea, and bilateral infiltrates on chest imaging (1, 23, 69). Edema and prominent proteinaceous exudates, vascular congestion, and inflammatory clusters with fibrinoid material and multinucleated giant cells has also been reported in lungs of COVID-19 infected patients (70).

Overall, the transcriptional footprint of SARS-CoV-2 infection is distinct from other highly pathogenic coronaviruses and common respiratory viruses such as IAV, HPIV3, and RSV. It is noteworthy that, despite a reduced IFN-I and -III response to SARS-CoV-2, recent studies show a consistent chemokine signature (71).

## Immune Response Against Coronavirus

In patients with COVID-19, the white blood cell count can vary between leukopenia, leukocytosis, and lymphopenia, although lymphopenia appears to be more common (1, 72). Importantly, the lymphocyte count is associated with increased disease severity in COVID-19 (73, 74). Lymphopenia and lower lymphocyte counts indicated a poor prognosis in COVID-19 patients (75, 73). ICU patients suffering from COVID-19 have lymphocyte counts of 800 cells/μl and a reduced chance for survival (23). The etiology and mechanisms of lymphopenia in COVID-19 patients is unknown but SARS-like viral particles and SARS-CoV RNA has been detected in T cells suggesting a direct effect of SARS virus on T cells potentially through apoptosis (74, 75, 76).

The role of DCs in the host defense against COVID-19 unclear. During infection with SARS-CoV, antigen-presenting cell (APC) function is altered and impaired DC migration results in reduced priming of T cells. This will lead to a fewer number of virus-specific T cells within the lungs (77, 78). After initial infection with virus, lung resident respiratory DCs (rDCs) seek out the invading pathogen or antigens from infected epithelial cells, and when activated, process antigen and migrate to the draining (mediastinal and cervical) lymph nodes (DLN). Once in the DLNs, rDCs present the processed antigen in the form of MHC/peptide complex to naïve circulating T cells. Engagement of the T cell receptor (TCR) with peptide-MHC complex and additional co-stimulatory signals induce T cell activation, vigorous proliferation and migration to the site of infection (79, 80).

Cytotoxic lymphocytes (CTLs) and natural killer (NK) cells are important for the control of viral infection, and the functional exhaustion of cytotoxic lymphocytesis may increase the severity of diseases. In patients with COVID-19, the total number of NK and CTLs are decreased which is in parallel with exhaustion of their function and upregulation of NK inhibitory receptor CD94/NK group 2 member A (NKG2A) (81). After successful recovery of COVID-19 patients, the number of NK and CD8+ T cells was restored with reduced expression of NKG2A. Furthermore, there is a lower percentage of CD107a + NK, IFN-γ^+^ NK, IL-2^+^ NK, and TNF-α^+^ NK cells in COVID-19 patients (81).

As indicated above, increased T cell apoptosis occurs in MERS infected patients (82, 83) and it is likely that this also happens in COVID-19 patients. Interestingly, the decreased number of CD4+ and CD8+ T cells in the peripheral blood of SARS-CoV-2-infected patients possess high proportions of HLA-DR (CD4 3.47%) and CD38 (CD8 39.4%) double-positive cells indicating highly activated cells (68). In addition, there was impaired activation of CD4 and CD8 cells evidenced by the appearance of CD25, CD28, and CD69 expression on these T cell subsets (84, 85). These factors may together account for the delayed development of the adaptive immune response and prolonged virus clearance in severe human SARS-CoV infection (86).

Decreased numbers of T cells strongly correlated with the severity of the acute phase of SARS disease in humans (87, 88). Both the S and N proteins of SARS-CoV contain immunogenic epitopes that are recognized by CD4 and CD8 T cells. Viral S protein induce neutralizing antibodies and immunization with vaccines encoding the virus N-protein able to induce eosinophilic response in animals (89). In order to produce neutralizing antibodies, it is important that the viral antigen is recognized by APC as these subsequently stimulate the body’s humoral immunity via virus-specific B and plasma cells (Figure 1). In SARS, IgM and IgG are important antibodies and the IgM antibody was detected in patient’s blood 3–6 days after infection and IgG could be detected after 8 days (90, 91). The SARS-specific IgM antibodies disappeared by the end of week 12, whilst the IgG antibody can last for a long time. This suggests that generation of IgG antibodies may be essential to provide a longer term protective role (92).

Understanding the immune response to SARS-CoV-2 is crucial for vaccine development. HLA class I and II epitope pools have been used to detect CD4+ and CD8+ T cells in 100 and 70% of convalescent COVID patients (93). The CD4+ responses to the SARS-CoV-2 Spike protein correlated with the magnitude of antiviral immunoglobulin titers although T cell responses were also found against M, N, and open viral proteins. Intriguingly, 40–60% of non-SARS-CoV-2 exposed individuals also possessed CD4 + cell responses against SARS-CoV-2 indicating a degree of cross-reactivity between CoVs (93).

In addition to cell-mediated and humoral-mediated defense by the immune system, pro-inflammatory cytokine release also helps against COVID-19 infection. Effector cytokines such as IFN-γ directly inhibit viral replication and enhance antigen presentation (94). However, it has been postulated that SARS-CoV-2, due to the secretion of a novel short protein encoded by orf3b, inhibits the expression of IFNβ and enhances viral pathogenicity (95). Chemokines produced by activated T cells recruit more innate and adaptive cells to control the pathogen burden. Cytotoxic molecules such as granzyme B directly kill infected epithelial cells and help eliminate the pathogen (96–99). One of the main mechanisms for ARDS induced by SARS-CoV-2 is the cytokine storm, the deadly uncontrolled systemic inflammatory response resulting from the release of large amounts of pro-inflammatory cytokines (100).

Besides lymphocytes, other innate immune cells also play a role in the pathogenesis of COVID-19. For example, neutrophils and neutrophil-associated cytokines such as CXCL2 and CXCL8 are elevated in the blood and serum of COVID-19 patients (101). This may have prognostic value for identifying individuals at risk for developing severe disease.

The cytokine storm syndrome (CSS) is the result of an immune system running wild. In this condition the regulation of immune cells is often defective, resulting in the increased production of inflammatory proteins that can lead to organ failure and death. Among these inflammatory mediators released by immune effector cells are the cytokines IFN-α, IFN-γ, IL-1β, IL-6, IL-12, IL-18, IL-33, TNF-α, and transforming growth factor (TGF)β and chemokines such as CCL2, CCL3, CCL5, CXCL8, CXCL9, and CXCL10 (1, 66, 86, 102). Early clinical (fever, confusion) and laboratory (blood hyperferritinemia, lymphopenia, prolonged prothrombin time, elevated lactate dehydrogenase, elevated IL-6, elevated C-reactive protein, elevated soluble CD25) results from critically ill COVID-19 patients suggest the presence of a CSS causing ARDS and multi-organ failure (23, 72, 103) as seen with SARS-CoV and MERS-CoV infection (68).

Secondary hemophagocytic lymphohistiocytosis (sHLH) is an under-recognized, hyperinflammatory syndrome which is accompanied by a fulminant and fatal hyper cytokinaemia with multi-organ failure which has been reported following viral infections (104) and occurs in 3.7–4⋅3% of sepsis cases (105). A cytokine profile resembling sHLH is associated with COVID-19 disease severity, characterized by increased IL-2, IL-7, GCSF, IP-10, MCP-1, and MIP-α (1). All patients with severe COVID-19 should be screened for hyperinflammation such as increased ferritin, decreased platelet counts and erythrocyte sedimentation rate (106) to identify the subgroup of patients for whom immunosuppression could improve mortality. Therapeutic options include steroids, intravenous immunoglobulin, selective cytokine blockade (e.g., anakinra or tocilizumab) and JAK inhibition (107–111) and the results are eagerly awaited.

Granulocyte macrophage colony-stimulating factor (GM-CSF) is an immunoregulatory cytokine with a pivotal role in initiation and perpetuation of many inflammatory diseases. GM-CSF links T-cell-driven acute pulmonary inflammation with an autocrine, self-amplifying cytokine loop that leads to monocyte and macrophage activation. This loop has been targeted in CSS and in chronic inflammatory disorders. Importantly, the expansion of GM-CSF-expressing CD4+ T cells (Th1), CD8+ T cells, natural killer cells, and B cells are associated with disease severity in COVID-19 patients (112).

It is plausible that GM-CSF serves as an integral link between the severe pulmonary syndrome-initiating capacity of pathogenic CD4+ Th1 cells (GM-CSF+ IFNγ+) with the inflammatory signature of monocytes (CD14 + CD16 + with high expression of IL-6) (113). The potential risks associated with inhibition of GM-CSF in the context of viral infection and the challenges of doing clinical trials in this setting, highlight the fact that the mechanism(s) of induction of the cytokine storm are not well understood and that unknown genetic factors might be playing a role.

The reason for the resistance of children to COVID-19 is also unclear. However, it seems that their immune reactivity is lower than in adults and that although infants are susceptible to SARS-CoV-2 infection the severity of the disease is generally low (114). In addition, other reports have hypothesized that the lower risk of infection among children is due to differential expression of angiotensin-converting enzyme 2 (ACE2) which increases its gene expression within nasal epithelial with age (115).

A genetic predisposition to infectious viral disease has been ascribed to young and healthy adults who succumb to SARS-CoV-2 infection with resultant overt symptoms of COVID-19. However, there is limited evidence available as yet to delineate any specific genetic markers. Dementia has been associated with an enhanced risk of COVID-19 susceptibility and higher mortality in United Kingdom patients. The apolipoprotein E (ApoE) e4 genotype is associated with an increased risk of dementia and Alzheimer’s disease. Interestingly, within the United Kingdom Biobank, ApoE e4e4 homozygotes were 2.3–4.0-fold more likely to be COVID-19 test positives (OR = 2.31, 95% CI: 1.65 to 3.24) and may relate to co-expression of ApoE e4 and ACE2 within type 2 alveolar epithelial cells (116). The risks for COVID-19 mortality were not associated with chronological age or age-related comorbidities. Further studies are needed to validate these results in another cohort and to understand the mechanisms linking ApoE genotypes to COVID-19 severity.

Furthermore, there is a global effort to define the human genetics of protective immunity to SARS-CoV-2 infection (117). The goal is to compare extremes of SARS-CoV-2 susceptibility in young individuals with very severe disease and subjects with no infection despite high viral exposure.

The presence of metabolic balance syndrome/obesity, and particularly its complications, such as diabetes and hypertension, is associated with an increased propensity to develop a more serious illness, requiring hospital admission and probably invasive ventilation (111). Furthermore, patients with previous cardiovascular metabolic diseases also have a greater risk of developing severe disease highlighting the fact that the presence of comorbidities greatly affects the prognosis of the COVID-19 (118). Whether there is a genetic link to this increased risk in Caucasians is unknown but such a link is present between COVID-19 and ACE2 polymorphisms in disorders such as diabetic mellitus, cardiac diseases in Asian populations (119, 120).

In conclusion, the host immune response is the critical factor in driving COVID-19 and analysis of this response may provide a clearer picture as to how the host response impacts upon the disease severity in some individuals while most infected people only show mild symptoms or no symptoms at all. Early analysis of blood samples using scRNA-seq has revealed some interesting features (121). These include a varied IFN-stimulated response and HLA class II downregulation. Interestingly, in subjects with acute respiratory failure requiring mechanical ventilation a novel B cell-derived granulocyte population was identified. Importantly, circulating leukocytes do not express high levels of pro-inflammatory cytokines and chemokines suggesting that the COVID-19 cytokine storm is driven by cells within the lung.

Thus, the study of the host immune response from acute and convalescent individuals will provide molecular insights into mechanisms by which we may enable protection and long-term immune memory and enable the design of prophylactic and therapeutic measures to overcome future outbreaks of similar coronaviruses.

## Author Contributions

EM wrote the original manuscript. PT, MV, GF, and IA revised the manuscript. All authors contributed to the article and approved the submitted version.

## Conflict of Interest

The authors declare that the research was conducted in the absence of any commercial or financial relationships that could be construed as a potential conflict of interest.

## Footnotes

1 https://www.worldometers.info/coronavirus/

2 https://www.who.int/news-room/q-a-detail/q-a-similarities-and-differences-covid-19-and-influenza
